# Quality of life of gay and bisexual men during emerging adulthood in Taiwan: Roles of traditional and cyber harassment victimization

**DOI:** 10.1371/journal.pone.0213015

**Published:** 2019-02-28

**Authors:** Huei-Fan Hu, Yu-Ping Chang, Chienho Lin, Cheng-Fang Yen

**Affiliations:** 1 Department of Psychiatry, Tainan Municipal Hospital (Managed by Show Chwan Medical Care Corporation), Tainan, Taiwan; 2 School of Nursing, The State University of New York, University at Buffalo, New York, United States of America; 3 Department of Psychiatry, Chimei Medical Center, Tainan, Taiwan; 4 Department of Psychiatry, Kaohsiung Medical University Hospital, Kaohsiung, Taiwan; 5 Graduate Institute of Medicine, and Department of Psychiatry, School of Medicine, College of Medicine, Kaohsiung Medical University, Kaohsiung, Taiwan; Hunter College, UNITED STATES

## Abstract

This study examined factors related to the quality of life (QOL) of gay and bisexual men during emerging adulthood in Taiwan. The factors included traditional harassment (e.g., verbal ridicule, relational exclusion, physical aggression, and theft of belongings), cyber harassment, sex- and gender-related factors (e.g., sexual orientation, age at initial identification of sexual orientation, self-reported level of gender nonconformity, and perceived social acceptance toward homosexuality and bisexuality), and demographic characteristics. In total, 305 Taiwanese gay and bisexual men, aged 20–25 years, were recruited. Their QOL, traditional harassment, and cyber harassment data were collected using the World Health Organization Questionnaire on Quality of Life: Short Form, School Bullying Experience Questionnaire, and Cyberbullying Experiences Questionnaire, respectively. In total, 60.3%, 34.4%, 28.2%, and 29.5% of the participants reported experiencing traditional harassment, cyber harassment, traditional harassment across multiple contexts, and harassment in multiple forms, respectively. Both traditional and cyber harassment were significantly associated with lower QOL. Individuals who experienced traditional harassment across multiple contexts and harassment in multiple forms had lower QOL in nearly all domains than did individuals who had experienced traditional harassment in a single context and harassment of a single form. However, the QOL did not significantly differ between individuals who had experienced traditional harassment in a single context and nonvictims nor between individuals who had experienced harassment of a single form and nonvictims. Lower education level, older age at initial identification of sexual orientation, higher perception of gender nonconformity, and lower perceived social acceptance toward homosexuality and bisexuality were significantly associated with lower QOL. Clinical and public health professionals should consider these factors when developing programs to enhance the QOL of gay and bisexual men.

## Introduction

Lesbian, gay, and bisexual (LGB) people are more likely to experience physical and mental illnesses and subsequent disability than are heterosexual people [1, 2]. The minority stress model proposed by Meyer [3] attributes the health disparities of LGB people to greater exposure to life stressors accompanying their minority status, including victimization, discrimination, stigmatization, expectation of rejection, and vigilance [3]. Quality of life (QOL)—defined as individuals’ perceptions of their position in life according to the context of the culture and value systems in which they live—differs from metrics for illness-related disabilities, because individuals may react differently to similar levels of disability [4, 5]. To promote a holistic approach toward health and health care, the World Health Organization (WHO) developed the Questionnaire on Quality of Life: Short Form (WHOQOL-BREF) to evaluate QOL [5]. The WHOQOL-BREF is based on four domains, namely physical health, psychological wellbeing, social relationships, and environmental domains [5]. The broad concept of QOL is a crucial part of a holistic view of health care [6].

LGB people have a lower level of general [7] and health-related [8] QOL than do heterosexual people. Discrimination, as a behavioral manifestation of stigma [9], has been found to account for the low QOL in LGB people [10, 11]. Harassment related to sexual orientation is a common form of discrimination toward LGB people [12]. Such harassment may occur with the purpose or effect of violating the dignity of an individual or of creating an intimidating, hostile, degrading, humiliating, or offensive environment. Considering its harmful effects on LGB people, harassment related to sexual orientation has been deemed unlawful in many countries, such as the United Kingdom [12]. Repeated harassment not only leads to chronic stress in LGB adolescents but also increases their perception of negative attitudes toward sexual minorities, potentially causing them to internalize sexuality-related stigma and in turn to form a negative attitude toward themselves [13]. Self-stigma may have negative impacts on individuals’ perceptions of their position in life and result in low QOL in the environmental domains. Repeated harassment may also compromise victims’ cognitive, regulatory, and social abilities related to psychological development [14] and therefore may be associated with low QOL in the domain of social relationships in LGB individuals. Furthermore, QOL reflects individuals’ perceptions in the context of the value systems in which they live [5]; consequently, people with low QOL may not strive for the rights they deserve and may thus experience negative health results and low QOL in the domains of physical health and psychological wellbeing. For instance, Zhu et al. [15] reported low psychological QOL in Chinese men who had unprotected anal intercourse with men, indicating that low psychological QOL may limit their motivation and self-efficacy of asking their sexual partners to do protection in anal intercourse. Assessing the QOL of LGB people is crucial for comprehending their health needs and allocating resources to provide assistance.

A detailed understanding of factors related to QOL may aid in developing strategies for improving the QOL of LGB people. As described, QOL results from an interaction between the individuals and the culture and value systems in which they live [5]; thus, both individual and contextual factors related to QOL should be examined. Several individual factors are related to the QOL of LGB people. For instance, low educational attainment is associated with a decreased physical and psychological health-related QOL in LGB people [16]. Low educational attainment may limit occupation choices and income and therefore link to poor health status. Compared with gays and lesbians, bisexual people have worse general health [17]. It is hypothesized that bisexual women and men experience significantly higher levels of internalized stigma and sexual identity concealment, and lower levels of social support than lesbians and gays [17]. Early development and disclosure of LGB identity may increase rejection and exclusion by intolerant families, peers, and teachers and cause psychological stress in gay and bisexual adolescents [18]. Gender-role nonconformity significantly increases these individuals’ risk of experiencing sexuality-related bullying, which may in turn compromise psychosocial adjustment in sexual minority populations [19]. Regarding contextual factors, the QOL of LGB people is higher in cultures with accepting attitudes toward these people than in cultures with restrictive attitudes [7]. Moreover, bullying victimization is associated with a lower QOL in LGB people [20].

Several topics regarding individual and environmental factors related to QOL in LGB people warrant further research. First, studies have examined the relationship of the QOL of LGB people with traditional harassment (e.g., verbal, social, and physical harassment), but not cyber harassment. Cyber harassment has emerged in the digital age [21]; it involves harassment through electronic means, such as social networking sites, e-mail, chatrooms, instant messaging, websites, online games, and text messaging [22]. The Internet offers LGB people a medium for communication that is computer-mediated and does not involve face-to-face interaction. Online activities may diminish the risk of traditional harassment encountered by LGB people during face-to-face interactions. However, almost half of LGB adolescents experience online peer victimization [23]. Cyber harassment increases victims’ risk of psychological problems [22, 24]. Given that the Internet is a critical life context for LGB people, the relationship between cyber harassment and QOL of LGB people is a serious issue warranting further investigation. Second, LGB youths may experience harassment not only at schools but also in other environments, such as tutoring centers and workplaces. Whether traditional harassment across multiple contexts (i.e., harassment occurring at school and in the workplace) exhibits variable effects on QOL compared with traditional harassment in a single context should be investigated. Moreover, whether a combination of traditional and cyber harassment (i.e., harassment in multiple forms) has adverse additive effects on QOL compared with traditional or cyber harassment alone warrants further analysis. Third, generic QOL encompasses multiple domains. Whether related QOL factors vary across QOL domains in LGB people has not been examined. Fourth, few studies have examined individual and environmental QOL factors simultaneously.

Emerging adulthood is a phase of the life between adolescence and complete adulthood during which adolescents become more independent and explore life possibilities [25]. Individuals who were victims of any type of homophobic bullying in childhood experience more severe depression, anxiety, and physical pain during emerging adulthood than do nonvictims [26]. Whether traditional and cyber harassment victimization is associated with low QOL among gay and bisexual people during emerging adulthood warrants further study. Moreover, gays and lesbians are more likely to be bullying victims than are heterosexual people [27]. However, gay and bisexual boys have a higher risk of encountering negative criticism than do lesbian and bisexual girls [28]. Thus, the present study focused on the experiences of gay and bisexual men during emerging adulthood.

This study examined QOL-related factors including traditional harassment (e.g., verbal ridicule, relational exclusion, physical aggression, and theft of belongings), cyber harassment, sexual and gender-role factors (e.g., sexual orientation, age at initial identification of sexual orientation, self-reported level of masculinity, and perceived social acceptance toward homosexuality or bisexuality), and demographic characteristics in gay and bisexual men during emerging adulthood in Taiwan. The following hypotheses were established. First, according to the theory of minority stress, experiences of traditional and cyber harassment are significantly associated with lower QOL in gay and bisexual men during emerging adulthood. Second, compared with those experiencing traditional harassment in single contexts and harassment of a single form, gay and bisexual men experiencing traditional harassment across multiple contexts or harassment in multiple forms exhibit lower QOL. Third, lower educational level, identifying as a gay man (compared with identification as a bisexual man), early identification of sexual orientation, lower perception of masculinity, and lower perceived social acceptance toward the LGB community in society are significantly associated with lower QOL.

## Materials and methods

### Participants

Participants were recruited through advertisements on the social media websites (e.g., Facebook), bulletin board systems, and homepages of five health promotion and counseling centers for the LGB and transgender (LGBT) community from July, 2016 to May, 2017. The advertisement was also mailed to the LGBT societies of 25 colleges. Individuals who responded to the advertisement received a face-to-face interview. Those exhibiting any cognitive deficits (e.g., intellectual disability, intoxication, or dementia due to or withdrawal from substance use) that could have that prevented them from understanding the study purpose or completing the questionnaires were excluded. In total, two responders were excluded due to intellectual disability, and 305 gay or bisexual Taiwanese men, aged 20–25 years, were recruited. Written informed consent was obtained from all participants before assessment. This study was approved by the Institutional Review Board of Kaohsiung Medical University Hospital (KMUHIRB-SV(II)-20160018).

### Measures

#### WHOQOL-BREF Taiwan version

The WHOQOL-BREF is used to evaluate health-related QOL and to conduct cross-cultural comparisons [5]. The WHOQOL Taiwan group adapted the WHOQOL-BREF for use in Taiwan [29]. The WHOQOL-BREF Taiwan version contains four domains, including seven physical health items (including activities of daily living, dependence on medical aids, energy and fatigue, mobility, pain and discomfort, sleep, and work capacity, e.g., “To what extent do you feel that physical pain prevents you from doing what you need to do?”), six psychological wellbeing items (including body image and appearance, negative feelings, positive feelings, self-esteem, personal beliefs, thinking, learning, memory, and concentration, e.g., “How much do you enjoy life?”), four social relationship items (including personal relationships, social support, and sexual activity, e.g., “How satisfied are you with your personal relationships?”), and nine environmental domain items (including financial resources, physical safety and security, health and social care, home environment, opportunities for acquiring new information and skills, opportunities for recreation/leisure activities, physical environment, and transport, e.g., “How safe do you feel in your daily life?”). Each item is assessed on a 5-point scale from 1 to 5. The WHOQOL-BREF Taiwan version has well-established validity and reliability [29]. The WHOQOL-BREF manual provides a method for converting raw scores to transformed scores between 4 and 20. The higher the scores on the WHOQOL-BREF Taiwan version are, the higher the respondent’s perceived QOL is for the past 2 weeks.

#### Chinese version of the School Bullying Experience Questionnaire

Six items from the self-reported Chinese version of the School Bullying Experience Questionnaire (C-SBEQ) were transformed to evaluate participants’ experiences of traditional harassment in the past year [30] in schools, workplaces, social situations outside school or work, and other situations, such as army service, as well as during general interactions with family and in public. Two forms of traditional harassment victimization were evaluated: (1) verbal ridicule and relational exclusion (three items for experiences of social exclusion, hurtful name-calling, and being spoken ill of; e.g., “How often have others spoken ill of you?”) and (2) physical aggression and theft of belongings (three items for experiences of physical abuse, forced work, and confiscation of money, daily supplies, and snacks; e.g., “How often have others beaten you up?”). The responses for these six items were graded on a 4-point Likert scale (0 = never, 1 = just a little, 2 = often, 3 = all the time). A study on C-SBEQ psychometrics revealed that the C-SBEQ has acceptable reliability and validity [30]. The Cronbach’s α of the scale for evaluating traditional harassment was.77. In the present study, participants who did not answer 0 for any item were identified as self-reported victims of traditional harassment. Four additional items assessing places where traditional harassment occurred, including schools, workplaces, clubs outside schools, and others places (e.g., army service), were evaluated.

#### Cyberbullying Experiences Questionnaire

Three items of the Cyberbullying Experiences Questionnaire were used to assess respondents’ experiences of cyber harassment in the past year [31]. The three items addressed experiences related to mean or hurtful comments, pictures, photos, and videos and online rumor-spreading through emails, blogs, social media (e.g., Facebook, Twitter, and Plurk), pictures, and videos (e.g., “How often have other students posted mean or hurtful comments about you through emails, blogs, or social media?”). The responses to these items were graded on a 4-point Likert scale, ranging from 0 (never) to 3 (all the time). The Cronbach’s α of the scales for evaluating cyber harassment victimization was.72. In this study, participants who did not answer 0 on any item were identified as self-reported victims of cyber harassment.

#### Demographic characteristics and sexual orientation

This study evaluated participants’ age, education level (high school or lower vs. college or higher), self-identified sexual orientation (bisexuality vs. homosexuality), age at initial identification of sexual orientation, self-reported level of gender-role conformity, and perceived social acceptance toward homosexuality or bisexuality. Level of self-reported masculinity was rated on a Likert scale, with scores ranging from 1 (extreme femininity) to 9 (extreme masculinity). Level of perceived social acceptance toward homosexuality or bisexuality was also rated on a Likert scale, with scores ranging from 1 (highly acceptable) to 9 (highly unacceptable).

### Procedure

All participants filled out the questionnaires in the research room. Research assistants explained the procedures and methods for completing the questionnaires to the participants individually. The research assistants also resolved any difficulties that the participants encountered while completing the paper-and-pencil questionnaires. Data analysis was performed using SPSS (version 20.0; SPSS Inc., Chicago, IL, USA).

### Statistical analyses

The association of age, level of education, sexual orientation, age at initial identification of sexual orientation, self-reported masculinity, perceived social acceptance toward homosexuality and bisexuality, and level of traditional and cyber harassment victimization with the four domains of QOL on the WHOQOL-BREF Taiwan version was first examined using Pearson’s correlation and t test and then examined using multiple regression analysis. In addition, traditional harassment occurring at two or more places was defined as traditional harassment across multiple contexts. Experiencing both traditional and cyber harassment was defined as experiencing harassment in multiple forms. The association of traditional harassment across multiple contexts and harassment in multiple forms with the four domains of QOL was also examined using multiple regression analysis wherein the effects of demographics and sexual orientation were controlled. A *p* of.05 was considered statistically significant for all tests.

## Results

Table 1 presents the demographic data and data on sexual orientation, self-reported levels of masculinity, harassment victimization, and four domains of QOL on the WHOQOL-BREF Taiwan version. In total, 184 (60.3%), 105 (34.4%), 94 (30.8%), and 90 (29.5%) participants reported experiencing traditional harassment, cyber harassment, traditional harassment across multiple contexts, and harassment in multiple forms, respectively.

**Table 1:** Demographic data, sexual orientation, self-reported masculinity, harassment victimization, and quality of life (N = 305).

	*n* (%)^a^	Mean (SD)	Range
Age (years)		23.1 (1.7)	20–25
Education level			
High school or lower	29 (9.5)		
College or higher	276 (90.5)		
Sexual orientation			
Bisexuality	78 (25.6)		
Homosexuality	227 (74.4)		
Age at initial identification of sexual orientation		13.7 (3.5)	4–22
Self-rated level of masculinity		5.8 (1.3)	2–9
Perceived low social acceptance toward homo/bisexuality		5.2 (1.7)	1–9
Harassment victimization			
Victims of traditional harassment	184 (60.3)		
Setting in which traditional harassment occurred			
Single context	90 (29.5)		
Multiple contexts	94 (30.8)		
Two contexts	86 (28.2)		
Three or more contexts	8 (2.6)		
Victims of cyber harassment	105 (34.4)		
Types of harassment			
Victims of harassment in a single form	109 (35.7)		
Victims of harassment in multiple forms	90 (29.5)		
Experienced both traditional harassment in multiple contexts & any type of harassment in multiple forms	55 (18.0)		
Only experienced harassment in multiple contexts	31 (10.2)		
Only experienced harassment in multiple forms	35 (11.5)		
Quality of life on the WHOQOL-BREF			
Physical		14.8 (2.3)	8–20
Psychological		12.8 (2.8)	5–20
Social relationships		13.7 (2.6)	6–20
Environment		14.1 (2.3)	7–20

^a^: denominator = 305

WHOQOL-BREF: World Health Organization Questionnaire on Quality of Life: Short Form Taiwan version

Tables 2 and 3 present the results of Peason’s correlation and *t* tests examining the relationships of age, level of education, sexual orientation, age at initial identification of sexual orientation, self-reported masculinity, perceived social acceptance toward homosexuality and bisexuality, and traditional and cyber harassment victimization according to the four domains of QOL on the WHOQOL-BREF Taiwan version. The results indicated that lower levels of peceived social acceptance toward homosexuality and bisexuality and traditional and cyber harassment victimization were significantly associated with lower QOL in all four domains, whereas higher levels of self-reported masculinity were significantly associated with higher QOL on all four domains. Participants with higher education levels (college or higher) had higher QOL in the physical, psychological, and environmental domains than did those with lower education levels (high school or lower). Earlier age at initial identification of sexual orientation was significantly associated with higher QOL in physical and social relationship domains.

**Table 2:** Relationships of age, age at initial identification of sexual orientation, masculinity, and social acceptance with QOL: Peason’s correlation.

	Physical		Psychological		Social relationships		Environment	
	*R*	*p*	*r*	*p*	*r*	*p*	*r*	*p*
Age	-.090	.117	-.083	.147	-.096	.093	-.058	.314
Age at initial identification of sexual orientation	-.153	.007	-.081	.160	-.126	.027	-.111	.053
Masculinity	.184	.001	.204	<.001	.132	.021	.171	.003
Social acceptance	-.172	003	-.156	.006	-.140	.014	-.070	.224

**Table 3:** Differences in QOL across education levels, sexual orientations, and experiences of traditional and cyber harassment: *t* test.

	Physical			Psychological			Social relationships			Environment		
	Mean (SD)	*t*	*p*	Mean (SD)	*t*	*p*	Mean (SD)	*t*	*p*	Mean (SD)	*t*	*p*
Education level												
Low	13.5 (1.8)	-3.167	.002	11.2 (3.0)	-3.470	.001	13.3 (2.5)	-.869	.385	12.3 (2.6)	-4.607	<.001
High	14.9 (2.3)			13.0 (2.7)			13.8 (2.6)			14.3 (2.2)		
Sexual orientation												
Bisexuality	14.7 (2.5)	-.396	.693	13.1 (2.9)	.912	.363	13.5 (2.6)	-1.106	.270	14.0 (2.2)	-.367	.714
Homosexuality	14.8 (2.2)			12.8 (2.7)			13.8 (2.5)			14.1 (2.3)		
Victim of traditional harassment												
No	15.4 (2.0)	4.310	<.001	13.3 (2.6)	2.470	.014	14.5 (2.4)	4.119	<.001	14.7 (2.0)	4.098	<.001
Yes	14.3 (2.3)			12.5 (2.8)			13.3 (2.6)			13.6 (2.4)		
Victim of cyber harassment												
No	15.2 (2.1)	4.527	<.001	13.2 (2.7)	3.015	.003	14.0 (2.5)	2.444	.015	14.5 (2.2)	4.962	<.001
Yes	14.0 (2.4)			12.2 (2.9)			13.2 (2.6)			13.2 (2.2)		

Table 4 lists the results of the multiple regression analysis for the association of demographic characteristics, sexual orientation, self-reported masculinity, and harassment victimization with QOL. The results indicated that later identification of sexual orientation; lower levels of education, self-reported masculinity, and perceived social acceptance toward homosexuality and bisexuality; and experience of cyber harassment were associated with lower QOL in the physical and psychological domains. Furthermore, experience of traditional harassment was associated with lower QOL in the physical domain. Later identification of sexual orientation and experience of traditional harassment were associated with lower QOL in the social relationship domain. Later identification of sexual orientation, lower education and self-reported masculinity levels, and experiences of cyber harassment were associated with lower QOL in the environmental domain.

**Table 4:** Associations of demographic data, sexual orientation, self-reported masculinity, and harassment victimization with QOL.

	Physical			Psychological			Social relationships			Environment		
	Beta	*t*	*p*	Beta	*t*	*p*	Beta	*t*	*P*	Beta	*t*	*p*
Age	-.056	-1.052	.294	-.050	-.918	.359	-.069	-1.240	.216	-.035	-.656	.512
Education	.135	2.533	.012	.164	2.982	.003	.019	.341	.733	.210	3.922	<.001
Sexual orientation	-.020	-.356	.722	-.066	-1.168	.244	.025	.435	.664	-.009	-.161	.872
Age at initial identification of sexual orientation	-.170	-3.118	.002	-.114	-2.030	.043	-.120	-2.097	.037	-.132	-2.407	.017
Self-rating level of masculinity	.153	2.848	.005	.171	3.096	.002	.108	1.920	.056	.146	2.699	.007
Perceived social acceptance toward homo/bisexuality	-.139	-2.628	.009	-.130	-2.382	.018	-.108	-1.946	.053	-.043	-.803	.423
Victims of traditional harassment	-.119	-2.060	.040	-.045	-.748	.455	-.171	-2.824	.005	-.101	-1.740	.083
Victims of cyber harassment	-.188	-3.293	.001	-.132	-2.236	.026	-.070	-1.166	.245	-.206	-3.601	<.001
Adjusted R^2^	0.161			0.109			0.079			0.155		

Table 5 presents the results of the multiple regression analysis on the association of QOL with traditional harassment across multiple contexts and harassment in multiple forms. The results indicated that compared with nonvictims, gay and bisexual men who experienced traditional harassment across multiple contexts had lower QOL in all four domains. Compared with those who experienced traditional harassment in a single context, gay and bisexual men who experienced traditional harassment across multiple contexts had lower QOL in all four domains. Moreover, compared with individuals who had not been victims of any harassment, the victims of harassment in multiple forms had lower QOL in all domains. Compared with the victims of harassment of a single form, those of harassment across multiple forms had lower QOL in all domains, except the social relationship domain. However, no significant difference in QOL was observed between single-context harassment victims and nonvictims and between single-form harassment victims and nonvictims.

**Table 5:** Associations of traditional harassment across multiple contexts and harassment in multiple forms with quality of life^a^.

	Physical			Psychological			Social relationships			Environment		
	Beta	*t*	*p*	Beta	*t*	*p*	Beta	*t*	*p*	Beta	*t*	*p*
Number of places in which traditional harassment occurred												
Single context vs. no victimization	-.119	-1.851	.066	-.029	-.442	.659	-.122	-1.823	.070	-.068	-1.032	.303
Multiple contexts vs. no victimization	-.350	-5.446	<.001	-.230	-3.392	.001	-.317	-4.729	<.001	-.305	-4.585	<.001
Multiple contexts vs. single context	-.233	-3.224	.002	-.187	-2.558	.011	-.213	-2.874	.005	-.214	-2.998	.003
Number of forms of harassment victimization												
Single form vs. no victimization	-.088	-1.335	.183	-.035	-.521	.603	-.119	-1.745	.082	-.064	-.949	.344
Multiple forms vs. no victimization	-.323	-4.898	<.001	-.181	-2.600	.010	-.246	-3.476	.001	-.337	-5.165	<.001
Multiple forms vs. single form	-.233	-3.514	.001	-.151	-2.211	.028	-.132	-1.910	.058	-.254	-3.858	<.001
Traditional harassment across multiple contexts and harassment in multiple forms												
Both vs. only multiple contexts	-.220	-2.078	.041	-.136	-1.244	.217	-.184	-1.699	.093	-.231	-2.198	.031
Both vs. only multiple forms	-.205	-1.981	.051	-.179	-1.703	.092	-.322	-3.219	.002	-.152	-1.547	.126

^a^: Controlled for the effects of demographic data and sexual orientation

Compared with the victims of only traditional harassment across multiple contexts but not harassment in multiple forms, those of both traditional harassment across multiple contexts and harassment in multiple forms had lower QOL in the physical and environmental domains. Compared with the victims of harassment in multiple forms but not in multiple contexts, those of both traditional harassment across multiple contexts and harassment in multiple forms had lower QOL in the social relationship domain.

## Discussions

The current results revealed that a high proportion of gay and bisexual men experienced traditional and cyber harassment during emerging adulthood. Both traditional and cyber harassment were significantly associated with lower QOL in certain domains. Compared with traditional harassment in a single context and harassment of a single form, traditional harassment across multiple contexts and harassment of multiple forms were more significantly associated with lower QOL in most domains for gay and bisexual men, respectively. Lower education level, older age at initial identification of sexual orientation, lower self-reported masculinity, and perceived lower social acceptance toward homosexuality and bisexuality were significantly associated with lower QOL in various domains.

### Harassment victimization and QOL

In a study in Taiwan, 87% and 40.2% of gay and bisexual men reported experiencing homophobic traditional and cyber harassment, respectively, in childhood and adolescence [32]. In the current study, 60.3% and 34.4% of gay and bisexual men, respectively, reported experiencing traditional and cyber harassment during emerging adulthood. The results indicated that harassment victimization was prevalent in the young adulthood experiences of gay and bisexual men. Moreover, traditional harassment was significantly associated with lower QOL in the physical and social relationship domains, and cyber harassment was significantly associated with lower QOL in the physical, psychological, and environmental domains. To conceptualize processes leading to LGB health disparities, Meyer articulated the theory of minority stress, which posits that increased stress due to discrimination, stigma, and prejudice faced by sexual minorities worsens their mental and physical health outcomes and increases their likelihood of using maladaptive coping strategies [3]. The label of a LGB community member increases the risk of harassment victimization, which partially accounts for health disparities in LGB population. According to the psychological mediation framework proposed by Hatzenbuehler [14], harassment victimization may exacerbate general emotional dysregulation, social and interpersonal problems, and cognitive processes that increase the risk of lower QOL. Moreover, the harassment victimization experienced by LGB people increases this group’s rate of risky health-related behaviors, such as alcohol and drug abuse [33, 34], which may further compromise LGB people’s physical and psychological QOL.

In this study, cyber, but not traditional, harassment victimization was significantly associated with lower QOL in the environmental domain. For gay and bisexual men, the Internet may be one of the most crucial life environments in early adulthood because it may serve as a crucial channel for them to manage their daily affairs and avoid face-to-face harassment. Compared with traditional harassment perpetrators, cyber harassment perpetrators can remain virtually anonymous [35]. In digital harassment, cruel and malicious behavior is easy to engage in, because the physical distance between the offender and the victim can be large [36]. The characteristics of cyber harassment may make gay and bisexual men feel unsafe, even in environments outside the Internet, further compromising their environmental QOL. By contrast, gay and bisexual men who perceive low environmental QOL may spend time online more frequently than those who perceive high environmental QOL. The risk of cyber harassment may increase as the time spent on online activities increases. The current study results should serve as a reminder to mental health and education professionals regarding the necessity of evaluating whether an individual is being bullied in the face-to-face context when these professionals address cyber harassment victimization among sexual minority youths.

A longitudinal study found that harassment victimization in adolescence leads to psychological distress during early adulthood among LGB people [33]. Therefore, the occurrence of cyber harassment should be monitored beginning in adolescence. However, research found that perpetrators of traditional harassment often perpetrate cyber harassment, indicating that cyber harassment may be an extended form of traditional harassment [36]. Furthermore, because cyber harassment is less detectable by adults than traditional bullying [37] and most cyber harassment victims do not report such bullying to an adult or use digital tools to prevent online incidents [38], experiences of traditional bullying may be used as an indicator for detecting the occurrence of cyber harassment among sexual minority adolescents.

### Traditional harassment across multiple contexts and harassment of multiple forms

In this study, over one-fourth of the gay and bisexual male respondents reported experiencing traditional harassment across multiple contexts (28.2%) and harassment in multiple forms (29.5%). Gay and bisexual men who encountered traditional harassment across multiple contexts and harassment in multiple forms had lower QOL in nearly all domains than did those who encountered traditional harassment in a single context and harassment in a single form, respectively; however, QOL did not significantly differ between victims of traditional harassment in a single context and nonvictims and between victims of harassment in single form and nonvictims. The victims of both traditional harassment across multiple contexts and harassment in multiple forms had lower QOL in the physical and environmental domains compared with those of only traditional harassment across multiple contexts and in the social relationship domain compared with those of only harassment in multiple forms. Victimization through both traditional harassment across multiple contexts and harassment in multiple forms indicate discrimination toward sexual minorities from multiple sources; during emerging adulthood, gay and bisexual men may perceive more severe marginalization than those who experienced harassment at a single context or form. Multiple marginalization indicators may interact synergistically and affect the health status of gay and bisexual men [39]. The current results indicated that cyber harassment may interact synergistically with traditional harassment, further compromising the QOL of gay and bisexual men.

### Age at initial identification of sexual orientation and QOL

A person’s development and disclosure of their LGB identity in childhood or adolescence may increase their exposure to peer discrimination and victimization in school [19]. The process of coming out to others for the first time is not easy for gay and lesbian adolescents and thus may cause them considerable stress [40], potentially leading to suboptimal developmental outcomes, including decrements in school performance, self-esteem, and physical and mental health [19, 38]. Research found that early coming out predicts suicidality during emerging adulthood in gay and bisexual men [41]. Contrary to the hypothesis that early identification of sexual orientation is significantly associated with lower QOL, the results of the current study found that an initial identification of sexual orientation at an older age is significantly associated with lower QOL. One of the possible etiologies accounting for the discrepancy between the hypothesis and the result is that the present study examined a participant’s age at initial identification of sexual orientation but not that at initial disclosure of sexual orientation to others. People may identify as LGB early in their lives but not disclose to others for the fear of being subjected to homophobia-motivated harassment. Earlier reporting of same-sex attraction is associated with a greater initial deficit in psychological wellbeing but a more rapid recovery [42]. Compared with those with later identification of sexual orientation, the gay and bisexual men with earlier identification may have recovered from initial QOL deficits related to sexual orientation during their emerging adulthood. Moreover, earlier identification of sexual orientation could signify greater social acceptance of sexual minorities. With greater perceived acceptance, sexual minority adolescents may be less likely to question or experience internal conflict regarding same-sex-oriented feelings or attraction [43].

### Gender nonconformity, perceived social acceptance toward homosexuality and bisexuality, and QOL

This study revealed that lower self-reported masculinity and perceived social acceptance toward homosexuality and bisexuality were significantly associated with lower QOL in several domains. The QOL of LGB people was higher in cultures with accepting attitudes toward homosexuality and bisexuality than in cultures with restrictive attitudes [7]. Given that gender nonconformity and nonacceptance of the LGB community significantly increases the risk of harassment victimization in this community [38], harassment victimization may account for the association of gender nonconformity and nonacceptance of the LGB community with lower QOL in this community. The present study revealed that, even after controlling for the effects of harassment victimization, a lower perception of masculinity and nonacceptance of LGB people within a society remained significantly associated with lower QOL. An endorsement of a heteronormative culture contributes to the extent of homophobic harassment directed toward LGB people [39]. Gay and bisexual men who perceive lower levels of masculinity may be rejected by members of a heteronormative culture during their childhood, adolescence, and emerging adulthood. The negative effects of feeling rejected by a heteronormative culture may compromise these men’s emotional regulation and social skills, both of which are essential to a good QOL. A longitudinal study found that childhood bullying victimization is associated with a lack of social relationships, economic hardship, and poor perceived QOL at 50 years of age [44], indicating the long-term negative effects of peer maltreatment from childhood.

### Implications

The present study found several modifiable and unmodifiable factors related to QOL in gay and bisexual men. Traditional and cyber harassment, especially traditional harassment across multiple contexts and harassment in multiple forms were significantly associated with low QOL in several domains among gay and bisexual men during emerging adulthood. The results suggest that prevention programs for harassment of gay and bisexual men should be implemented as early as possible. Schools should provide LGBT students the critical resources, including gay-straight alliances and supportive educators [45]. Schools should also provide all students the curriculum to educate them the concepts of sexual orientation equality [45]. Comprehensive bullying/harassment policy is also essential to effectively promote positive school climate and individual student well-being, including feelings of safety, achievement, and positive mental health [46]. Early detection of and intervention for harassment victimization are necessary at not only schools but also workplaces, military bases, and any environment where young adult men may gather. Gay and bisexual men who report harassment victimization in one context should be asked whether they have encountered harassment in other contexts. Similarly, the experience of cyber harassment should be clarified among those who experience traditional harassment.

Mental health professionals should be familiar with various models of intervention for harassment victimization of gay and bisexual men. Basically, mental health professionals can counsel LGB people by helping them identify accepting individuals in their lives who can provide them with support. Mental health professionals can advise LGB people on how to react to being harassed and encourage them to join safe, supportive networks to reduce social isolation [47]. Furthermore, mental health professionals should know the psychological constructs related to minority stress, such as rumination, rejection sensitivity, and perceived burdensomeness that have implications for approaches to LGB-affirmative mental health intervention [46]. Research showed that young gay men’s psychosocial functioning was improved through expressive writing that targeted gay-related stress [48]. Research also found that adapted cognitive-behavioral intervention focusing on stigma-related stressors could decrease depressive symptoms, alcohol use, sensitivity to rejection, internalized homophobia, and rumination, as well as increase emotional regulation, perceived social support, and assertiveness [49]. How traditional harassment across multiple contexts and harassment of multiple forms compromise the QOL of gay and bisexual men warrants further investigation along with the development of QOL improvement programs.

The present study also found that lower education level, older age at initial identification of sexual orientation, higher perception of gender nonconformity, and lower perceived social acceptance toward homosexuality and bisexuality were significantly associated with lower QOL. Although these factors are not modifiable, they can serve as the indicators for clinicians and public health professionals to develop subgroup-specified prevention and intervention programs for harassment in gay and bisexual men.

### Limitations

This study involved several limitations. First, the data were exclusively self-reported, and we did not obtain additional information; this use of a single data source may have resulted in shared-method variances. Second, the present study only included young gay and bisexual men; therefore, the result may not be generalizable for the whole LGBT community. However, long-term exposure to homophobia-motivated harassment also significantly accounts for poor general health, disability, and depression among older LGB adults [18]. Third, because of the cross-sectional nature of this study, conclusions could not be drawn regarding the causal relationship between bullying victimization experiences and lower QOL. Fourth, the present study surveyed general experiences of harassment victimization but did not focus on homophobia-motivated harassment victimization.

## Conclusions

This study revealed that both traditional and cyber harassment is prevalent among gay and bisexual men during emerging adulthood. Individual factors, such as education level, age at initial identification of sexual orientation, self-reported masculinity, and interacting individual–environment factors, such as traditional and cyber harassment and perceived lower social acceptance toward homosexuality and bisexuality, were significantly associated with lower QOL. Clinical and public health professionals should consider these factors, particularly traditional harassment across multiple contexts and harassment in multiple forms, when developing programs to enhance the QOL of gay and bisexual men.

## Supporting information

S1 Database SPSS database of qualitative responses to survey questions. (SAV)
